# Decoding the role of gut mycobiota in immune regulation and disease

**DOI:** 10.1080/21505594.2025.2541704

**Published:** 2025-08-17

**Authors:** Tiantian Zhang, Feifan Guo, Cihan Zhang, Youqun Xiang

**Affiliations:** a Operating Room, Hospital of Wenzhou Medical University, Wenzhou, Zhejiang, China; b Department of Colorectal and Anal Surgery, Hospital of Wenzhou Medical University, Wenzhou, Zhejiang, China

**Keywords:** Gut mycobiota, fungal-host interactions, immune regulation, fungal dysbiosis

## Abstract

The gut mycobiota, a minor but influential component of the microbiota, is increasingly recognized for its role in shaping host immunity and influencing health. Advances in sequencing technologies have now highlighted the diversity and complexity of these fungal communities. Dysbiosis of the gut mycobiota is associated with various diseases, including inflammatory bowel disease (IBD) and asthma, through mechanisms involving fungal-host interactions, immune modulation, and cross-reactivity. Despite these insights, the mechanisms underlying fungal–immune interactions remain incompletely understood. In this review, we explore the composition of the gut mycobiome and its role in health and disease, focusing on its interaction with the host immune system. We also explore the therapeutic potential of targeting the mycobiome to mitigate disease outcomes. By integrating recent findings, this review aims to deepen the understanding of fungal contributions to immune homoeostasis and modulating the mycobiome in the context of health and disease.

## Introduction

The human gut hosts a diverse community of microbes, collectively referred to as the gut microbiota, which plays a crucial role in shaping host immunity and influencing both health and disease. While bacteria are the dominant members of the gut microbiota, the mycobiota which comprising various fungi, represents a minor but significant component of the gut microbiome. These fungi interact with both bacterial communities and the host, influencing physiological processes and contributing to the development or prevention of pathological conditions. Commensal fungi residing at barrier surfaces have been increasingly recognized for their role in regulating tissue homoeostasis and enhancing resistance to infectious and immune-mediated diseases [1].

Among immune-mediated disorders, inflammatory bowel disease (IBD) and asthma have emerged as prominent models for studying fungal–immune interactions. Recent advances highlighting the gut-lung axis – a bidirectional communication network linking the gastrointestinal and respiratory systems via microbial metabolites, shared immune responses, and trafficking of immune cells. Both IBD and asthma are chronic inflammatory diseases that exhibit mucosal immune dysregulation, involving similar immune pathways such as Th17 and type 2 innate lymphoid cells (ILC2s) [2,3].

The interaction between the fungal microbiota and the immune system has profound implications for understanding and treating immune-mediated conditions, including inflammatory bowel disease (IBD) and asthma [2,3]. In IBD, overgrowth of fungi disrupts gut homoeostasis, exacerbating inflammation through impaired antifungal immunity and T_H_17-mediated responses [4–7]. Similarly, fungal dysbiosis in the gut has systemic effects, promoting allergic inflammation in the lungs through fungal metabolites and immune cell trafficking [3,8–10]. These findings highlight the intricate interplay between gut fungi and host immunity, supporting the notion that gut mycobiota play roles beyond local gut effects, making IBD and asthma ideal case studies to investigate mycobiota-mediated immune modulation.

Therapeutic strategies targeting the gut mycobiota are emerging as promising approaches to restore gut homoeostasis and ameliorate disease outcomes [11]. However, it remains challenging due to the complex interactions between fungi, bacteria, and the host immune system. In this review, we explore the structural and functional features of the gut mycobiota and its role in regulating local and systemic immunity, highlighting the potential involvement of fungi and fungal-sensing immune pathways in disease aetiology. By decoding the interplay of fungal microbiota and host immunity, we also discuss strategies for modulating the mycobiome to restore immune homoeostasis and improve patient outcomes.

### Decipher the composition of mycobiome in the gut

Before the development of advanced next-generation sequencing, the intestinal fungal were identified by selective culture methods which result in a lot information loss. The development of culture-independent methods such as PCR and next-generation sequencing technologies are making large-scale microbiome projects more feasible. These analyses have shown that the *Candida* genera typically dominate the intestinal fungal community in mice and humans. *Saccharomyces, Malassezia, Penicillium, Cladosporium, Aspergillus, Debaryomyces, Pichia, Cladosporium*, *Galactomyces and Trichosporon* are also the most prevalent fungal genera in the human gut based on ITS2 and 18S rRNA sequencing [12].

#### Fungal communities in the human intestine

The fungus in the faecal samples is usually used to represent the gut mycobiome. Human Microbiome Project (HMP) was founded to characterize the “healthy” human microbiome as a baseline for reference and comparison studies. A compilation of 36 studies ranging from 1917 to 2015 have reported 267 distinct fungi species. Among them, 75% of the species were exclusive for only one study, and only 15 were reported in 5 or more studies [13]. This study indicates that though many fungal species have been observed in different studies, only a few are widely distributed in the human gut. To gain further insight into the variability and stability of the mycobiome, a longitudinal study consisting of 317 samples from 147 volunteers was conducted using samples from Human Microbiome Project stool. The results indicate that fungal diversity is lower than bacterial diversity in the gut, and that yeast genera such as *Saccharomyces*, *Malassezia*, and *Candida* are the dominant species. Except these common species, not only inter- but also intra-volunteer variability were high [12]. A recent study identified a total of 12,453 fungal isolates from 135 faecal samples of healthy people. After whole-genome sequencing, a total of 760 genomes, covering 206 fungal species, were assembled, of which 69 were identified for the first time. A further comparison of CGF with human fungal strains from the respiratory tract, digestive tract, urogenital tract and skin in the NCBI database revealed that only 32 common strains existed, which greatly expanded the number of available species of fungi in culture [14].

#### Fungal communities in the mice intestine

Due to the different sources of food and breeding environment, there exist high variability and transient instability in the gastrointestinal mycobiota of laboratory mice compared to human fungal microbiota [15–17]. To define the mouse intestinal fungal microbiome, a collection of 23 mice (wild-type or dectin1^−/−^) samples were sequenced. Over 100 different well-annotated fungal species representing at least 50 genera were identified which suggested a high variability and diversity of mouse mycobiome. The food-borne fungi only made up to 1.5% of total fungi identified in the mouse intestine. Whereas 97.3% sequences belong to only 10 fungal species, and *Candida tropicalis* represent 65.2% of them. This suggests that though with high diversity, most of the commensal fungi exist indigenous to the mouse gut [18]. Although sequencing data suggest fungi to be a component of gut microbiome, it still remains uncertain which fungal species can truly adapt to the mouse gastrointestinal tract. *Kazachstania pintolopesii* has been identified to be an infectious agent in wild urban and rural mice [19]. Recently, it has been shown that *Kazachstania pintolopesii* has an exceptional ability to colonize the mouse gastrointestinal tract and dominate the gut mycobiome independent of gut bacteria [20].

### The role of gut fungi and disease susceptibilities

The role of the intestinal mycobiome in health and disease has garnered significant attention in recent years, largely due to growing evidence that the gut mycobiota can influence not only intestinal health but also a variety of other systemic conditions. Except the straightforward connection between the gut fungi and intestinal health [2], the gut fungi are also involved in regulating peripheral immune responses such as asthma and carcinogenesis [3,21].

#### Immunological interaction between fungi and host immunity

The fungi are highly immunologically reactive and can influence both local and systemic immune responses. Previous reviews have systematically examined host immune responses to fungal pathogens [22,23], intestinal immunity against gut fungi remains understudied. However, certain mechanisms identified in systemic antifungal immunity may also apply to intestinal barrier defence (Table 1). Key mechanisms include pattern recognition receptor (PRR)-mediated pathways, such as those involving Toll-like receptors (TLRs), C-type lectin receptors (CLRs). Fungal cell wall components – including β-glucan, zymosan, mannan, and chitosan – as well as fungal secreted proteins can be sensed by PRRs or intracellular mechanism (e.g. TLR2, TLR4, Dectin-1, Dectin-2), and elicit immune responses or interfere with cell intrinsic signalling. CLRs has been identified to be essential for anti-fungal immunity. The fungal cell wall components (the most abundant fungal components) will bind to CLRs to stimulate Syk tyrosine kinase and the adaptor protein CARD9, thereby activating the NLRP3 inflammasome and NF-κB signalling. These pathways drive cytokine production and ultimately amplify innate immune defences against fungal invasion. Notably, these receptors also direct and modulate the development of adaptive immunity, including T_H_1, T_H_2 and T_H_17 immune response. T_H_1 mediated type I responses are essential for the control of systemic fungal infections by IFN-γ production, leading to neutrophil recruitment, defensin production, phagocyte activation, and finally elimination of pathogenic fungi [22]. Type 2 inflammation encompasses both allergic and non-allergic responses characterized by eosinophil infiltration, T_H_2 cell activation, group 2 innate lymphoid cells (ILC2s), and the signature cytokines IL-4, IL-5, and IL-13 [24]. T_H_17 responses are primarily required for mucosa/barrier immunity. Importantly, T_H_17 cells that can be induced in the gut and mesenteric lymph nodes upon intestinal Candida colonization [22,25].

**Table 1.** Key immune components and cells in fungal recognition.

Immune components/cells	Type	Fungal Target/Ligand	Function/Role in Disease Context
Dectin-1 (CLEC7A)	Innate PRR	β-glucan (fungal cell wall)	Activates CARD9 signalling → Th17 differentiation; associated with IBD severity [18]
CARD9	Adaptor Molecule	Downstream of Dectin-1	Mediates antifungal immunity and cytokine signalling [6]
TLR2/4	Innate PRR	Sel1 (*C. albicans*) Cpl1 (*C. neoformans*)	Triggers macrophage immune response [26,27]
Dectin-2	Innate PRR	High-mannose ligands (e.g. *W. mellicola*)	Triggers allergic responses, especially in asthma [28]
CX3CR1^+^ Mononuclear Phagocytes (MNPs)	Innate cell	Recognize *C. albicans*	Orchestrate Th17 response; essential in fungal clearance in gut [29]
Th17 Cells	Adaptive T cell	Fungal antigens (*C. albicans*)	Protective at homoeostasis; exacerbate colitis when dysregulated [6]
ILC2s	Innate Lymphoid cell	Unknown fungal cues	Promote lung eosinophilia in fungal-induced asthma [9]

#### The role of gut fungi in inflammatory bowel disease

Inflammatory bowel disease (IBD), which include ulcerative colitis (UC) and Crohn’s disease (CD), is a chronic and recurrent inflammatory status that happens primarily in gastrointestinal tract. The occurrence and progression of Crohn’s disease and ulcerative colitis have been well documented to be linked to imbalances in the microbiome, particularly bacterial populations. Important progress has been made recent years that not only bacteria, there exist fungi dysbiosis in IBD patient compared with healthy controls [,^30^]. Both CD and UC patients are clearly affected by mycobiota dysbiosis and they often experiencing fungi outgrowth.

Back to 1990s, Anti-*Saccharomyces cerevisiae* antibodies (ASCA) were found to be significantly higher in sera of patients with CD, which suggests that the gut mycobiota may be involved in the pathogenesis of CD [,^31^]. The ASCA IgA and IgG positive rate is over 50% in patients with CD and less than 5% in patients with non-IBD colitis or healthy controls [32–34,,]. Genetic correlation studies revealed no direct link between ASCA level and genetic defect; However, elevated ASCA is observed in the early stage of the disease [,^35^]. Recent study revealed that these antibodies have cross-reactivity with several fungal antigens including *C. albicans* and *Aspergillus* and *Malassezia* species [5,,34]. Further study revealed that secretory IgA preferentially bind to the cell wall of *Candida albicans* hyphae form and has higher affinity to *C. albicans* virulence factors. Functional analysis showed that secretory IgA treatment limits the frequency of *Candida* hyphal morphogenesis and commensalism in vivo [7]. These results suggest that fungi-induced antifungal antibodies produced in the gut may play a regulatory role in maintaining intestinal fungal commensalism by selectively targeting and coating virulence-associated fungal morphotypes.

Except the elevated sera level of anti-fungal antibodies, the changes of fungal communities have also been characterized in IBD. Recent evidence has shown the enrichment of *Candida* and *Malassezia* species in CD patients, which has been validated account for 65% of the microbiota in mouse models present dual roles as both commensal and pathogen [5,18].

*Candida* spp. has been found, statistically significant, present higher amount in the faecal mycobiome of patients with IBD from diverse samples [,^30^,^36^,^37^,]. *C. albicans* exist as the most common species related to the active IBD disease status, but *C. tropicalis* and *C. parapsilosis* have also been reported [,^37^]. Single nucleotide polymorphisms (SNPs) in *CLEC7A* that encode Dectin-1 receptor and CARD9 that encode the downstream adaptor protein in mediating fungal recognition in humans have been associated with inflammatory bowel diseases. Dectin-1 is a C-type lectin receptor (CLR) that recognizes β-glucan in fungal cell walls and signals through Card9 to induce inflammatory responses and further contributes to T_H_1/T_H_17 differentiation. Dectin-1-deficient (*Clec7a* ^−/−^) mice exhibited more severe experimentally colitis than wild-type mice [18]. Moreover, sequencing data revealed that the abundance of *Candida* spp. increased, whereas non-pathogenic *Saccharomyces* decreased in *Clec7a* ^−/−^ mice, indicating that Dectin-1 deficiency might lead to altered composition of indigenous fungi and *Candida* species are consistently associated with the inflamed gut.

*C. albicans* has been reported to be a major inducer of T help 17 (T_H_17) immunity. Under homoeostatic conditions, T_H_17 cells play a role in maintaining protective immunity at barrier sites such as gut and skin. Dysregulated T_H_17 activation contributes to inflammation and tissue damage which may exacerbate the colitis progression. Notably, CD patients have higher frequencies of *C. albicans* reactive T_H_17 cells in correlate with the severity of intestinal inflammation [6]. Genetic ablation of CX3CR1 mononuclear phagocytes (MNP), which are the primary cells that responsible for *C. albicans* recognition, also exacerbate experimental colitis in mice. CX3CR1+ MNP also contributes to the development of antifungal T_H_17 response. Ablation of Syk signalling in CX3CR1+ MNP result in fungi outgrowth and impaired T_H_17 response [,^29^]. These data suggest that proper recognition of *C. albicans* and the maintenance of gut fungal homoeostasis are essential for the establishment of host-fungal equilibrium and overall gut health.

In summary, defects in antifungal immunity contribute to increased susceptibility to intestinal inflammation. The progression of the disease can be influenced by the composition of the gut mycobiota, even when the gut bacteriome remains unaltered [8]. Various mouse models have demonstrated both detrimental [4,5] and protective [,^38^] role of fungi in regulate intestinal inflammation. These results highlight the understanding of the fungal components of the intestinal microbiota, which could be further refined by incorporating further mycobiome sequencing in IBD studies.

#### The role of gut fungi in asthma

Beyond their localized effects within the intestine, accumulating evidence underscores the role of gut fungi in modulating systemic immune responses at distal sites, including the lungs. Asthma, a chronic inflammatory airway disease, is often exacerbated by inhalation of fungal species such as *Aspergillus*, *Penicillium*, and *Alternaria*, which are recognized as potent environmental triggers [,^39^]. In addition to their direct impact on the lung environment, gut microbiota dysbiosis caused by antibiotic treatment can lead to a reduction in gut bacterial populations and an overgrowth of fungi, such as *Candida*. Bacterial metabolites, such as short-chain fatty acids (SCFAs), have been shown to modulate airway inflammation [,^40^]. Although fungi constitute a minor fraction of the gut microbiome, they are significantly larger than bacteria and capable of producing a diverse array of bioactive molecules [,^41^]. It has been shown that overgrowth of *Candida* in the gut leads to the accumulation of prostaglandins, which can promote allergic inflammation by inducing M2 macrophage polarization in the lungs, thereby exerting a distal immunomodulatory effect [10]. Moreover, antifungal drug-induced dysbiosis of the gut mycobiome can lead to a depletion of *Candida* populations while promoting the proliferation of other fungal species, such as *Wallemia mellicola*, *Aspergillus amstelodami*, and *Epicoccum nigrum* [8]. Mouse models have demonstrated that oral gavage with *Wallemia mellicola*, *Aspergillus amstelodami*, and *Epicoccum nigrum*, or single colonization with *Wallemia mellicola*, increases susceptibility to house dust mite challenge and exacerbates allergic immune responses [8,,42]. Recent studies have identified Dectin-2 as a key mediator of allergic responses triggered by *Wallemia* species. The severity of eosinophilic airway inflammation induced by airway sensitization to house dust mites (HDM) was significantly reduced in Dectin-2 knockout mice [,^28^]. However, the precise mechanisms by which these fungi are detected by intestinal components and subsequently amplify lung sensitivity to allergen challenges remain to be fully elucidated. Li et al. demonstrated that CX3CR1^+^ mononuclear phagocytes (MNPs) play a critical role in detecting fungal dysbiosis in the gut. This sensing mechanism mediates the Th2 responses and eosinophil accumulation during lung allergic inflammation [24].

Innate lymphoid cells type 2 (ILC2s) have been identified as key contributors to type 2 immune responses in the lung. Studies have demonstrated that ILC2s are essential for initiating adaptive Th2 immune responses and for promoting elevated immunoglobulin E (IgE) titres in the lung following allergen exposure. Notably, this process is significantly impaired in mice deficient in ILC2s, highlighting their critical role in orchestrating allergic inflammation [,^43^]. It has been revealed that an association exists between *Candida* overgrowth in the gut and an increased population of type 2 innate lymphoid cells (ILC2s) in the lung [9]. However, there is still unknown regarding how fungi overgrowth leads to ILC2 accumulation in the upper airway. A recent study on protozoa revealed that *Tritrichomonas musculis* (*T. musculis*) promotes the trafficking of gut-derived ILC2s to the lungs through succinate production, leading to lung eosinophilia and exacerbated allergic airway inflammation [,^44^]. These results lead us to hypothesize that gut fungi may employ similar strategies to remotely modulate pulmonary immunity by acting as regulators within the local gut niche, thereby influencing the lung eosinophil niche and exacerbating allergic asthma.

Collectively, the relationship between gut mycobiota and immune-mediated diseases is complex, with evidence supporting both causal and consequential roles of fungal dysbiosis (Table 2). Considering the critical role of immune responses in fungi detection, immune dysregulation can lead to fungal dysbiosis. Chronic inflammation in IBD disrupts mucosal barriers, enabling *Candida* hyphal invasion and dysbiosis [7]. Anti-Saccharomyces cerevisiae antibodies (ASCA) in Crohn’s disease patients reflect immune responses to commensal fungi [,^34^]. On the other hand, oral gavage with *Wallemia mellicola* in mice amplifies allergic lung inflammation, demonstrating that specific fungi can directly initiate distal immune responses [8]. Similarly, *Candida albicans* colonization induces pathogenic Th17 cell expansion in Crohn’s disease patients, with levels correlating to clinical disease severity [6]. Critically, fungal-derived metabolites – such as prostaglandin E2 (PGE2) from *Candida* and purine metabolites from *Malassezia*—directly modulate host immune signalling, further solidifying fungi as active contributors to immune dysregulation. Notably, immune dysregulation and fungal dysbiosis are not mutually exclusive phenomena, but rather exist in a bidirectional, self-perpetuating cycle. In Clec7a^−^/^−^ mice, loss of Dectin-1 (a fungal sensor) leads to *Candida* overgrowth and exacerbates colitis, directly linking impaired antifungal immunity to inflammation [18]. Gut fungal dysbiosis (e.g. *Candida* overgrowth) primes lung ILC2s via CX3CR1^+^ mononuclear phagocytes, exacerbating asthma, while pulmonary inflammation may reciprocally alter gut immunity [24].

**Table 2.** Causality vs. Consequence in gut mycobiota-disease interactions.

Disease	Fungal Species	Mechanism	Direction
IBD	*Candida albicans*	Th17-driven inflammation	Bidirectional
Asthma	*Wallemia spp.*	Dectin-2-mediated eosinophilia	Causal
IBD	*Malassezia spp.*	Purine metabolism disruption	Causal
IBD	General mycobiota	IgA deficiency → fungal overgrowth	Consequential

### Modulating the gut mycobiota for therapeutic applications

The gut mycobiome plays a critical role in shaping host immune physiology, significantly impacting disease development and progression. Pathogenic fungal infections pose a significant global health challenge, contributing to high morbidity and mortality. Recent studies suggest that the host innate/adaptive immune system can mitigate the harmful effects of pathogenic fungi in the gut, promoting their commensal fitness and supporting intestinal homoeostasis in a healthy state. Increasing evidence highlights the potential of targeting gut mycobiota as a promising therapeutic strategy for immune-mediated diseases (IMDs).

#### Treatment strategies differ for patients with vs. Without fungal dysbiosis

In patients with confirmed fungal overgrowth, such as *Candida albicans, Cryptococcus neoformans*, and *Aspergillus fumigatus* could present a threat on immunocompromised individuals [,^45^]. **Antifungal drugs** are generally present as the first choice to clear the pathogens for susceptible and immunocompromised individuals. Fluconazole treatment was found to be effective in alleviate the symptoms of ulcerative colitis patient colonized with *C. albicans* [,^46^]. However, prolonged antifungal treatment may cause microbiota changes of both fungi and bacteria. It may also cause undesired inflammatory disease outcome such as increased severity in acute and chronic models of colitis and asthma sensitization [8]. Conversely, in patients without signs of fungal dysbiosis, standard treatments targeting bacterial dysbiosis, immune suppression, or inflammation control may be sufficient. In such cases, antifungal therapy may be unnecessary and potentially harmful, as it could disrupt commensal fungi and impair mucosal immunity, worsening disease progression. Therefore, diagnostic tools are essential to distinguish fungal-associated IBD/asthma from non-fungal-driven cases, such as faecal mycobiome profiling, serum ASCA levels, and fungal qPCR. Also, Novel immune-based diagnostics strategies may benefit from the existing genetic polymorphism.

- Dectin-1: Mutations or polymorphisms in Dectin-1 (*CLEC7A*) have been associated with increased susceptibility to colitis.
- CARD9: Card9 deficiency results in impaired cytokine responses and has been linked to severe fungal infections and IBD-like phenotypes.

Therefore, recognizing this distinction may pave the way for precision medicine approaches, optimizing therapeutic interventions based on individual fungal signatures.

**The use of probiotics** has emerged as a potential alternative antifungal therapy. Bacterial strains such as *Lactobacillus* spp. and *Bifidobacterium* spp. have demonstrated antifungal activity in vitro. Oral administration of *Escherichia coli* strain Nissle 1917 (EcN), *Lactobacillus acidophilus*, *Bifidobacterium lactis*, *Bifidobacterium longum*, and *Bifidobacterium bifidum* has been shown to inhibit the growth of pathogenic fungi like *Candida albicans* in the gastrointestinal tract [,^47^,,,^48^]. Additionally, fungal species such as *Saccharomyces cerevisiae* and *Saccharomyces boulardii* are widely used probiotic yeasts for treating diarrhoea. These probiotics can modulate the mycobiota through various mechanisms, including the inhibition of filamentation [,^49^], adhesion [,^50^] and biofilm [,^49^,,^51^], all of which are critical virulence factors of pathogenic fungi. *Saccharomyces boulardii* have now been proved to be effective in treatment of gastrointestinal infections caused by *Helicobacter pylori, Salmonella*, and *Clostridium difficile* [52–54]. The underlying mechanisms by which *Saccharomyces boulardii* promotes gut health remain unclear. However, one study demonstrated that its modulatory effects depend on the presence of *Enterobacteriaceae*, suggesting that inter-kingdom interactions are critical for maintaining intestinal homoeostasis [,^55^]. Another possible mechanism is *S. boulardii* can support barrier function and regeneration of damaged intestinal tissue [,^56^]. However, similar to antifungal drug treatments, the use of *S. boulardii* should be approached with caution in individuals with weakened immune systems and older adults, as it may increase the risk of fungal infections [,^57^].

**Diet** has also been shown to influence the composition of the gut microbiota and mycobiota. Consumption of carbohydrate-rich diets has been shown to be associated with enhanced *Candida* growth [,^58^]. Western diets, which are high in fat and sugar and low in vegetable fibre have been shown to support the growth of *C. albicans* and decrease in beneficial anaerobic bacteria populations such as *Bifidobacterium*. It has been linked to the impairment of gut microbiota and development of a chronic, low-inflammatory state and disrupted gut barrier [,^59^]. Dietary sources of short-chain fatty acids (SCFAs), generated by gut bacteria, can inhibit the growth of *Candida* spp. by regulating its metabolism, gene expression, and signalling pathways [,^60^]. Not only the nutrients can affect the gut mycobiota, the food is also the important source of gut fungi. Yeast cells are the most common microorganism obtained from fermented food products such as cheese, bread and beer [,^61^]. *Wallemia mellicola*, a species correlated with asthma sensitization, have been detected in salt fermented products [,^62^]. Consumption of bread and beer have been shown to correlate with the amount of *Saccharomyces cerevisiae* in human stools [63]. plant-based diet and animal-based diet are also correlated with *Penicillium* and *Candida* abundance respectively [,^64^].

**Faecal microbiome transplantation (FMT)** refers to the clinical use of faecal suspension from healthy donors, administered into a patient’s gastrointestinal tract to restore intestinal microbiota. It was first found to be highly effective for treating *Clostridioides difficile* infections [,^65^]. Compared to probiotic treatments, FMT has also demonstrated superior effectiveness in restoring gut microbiota following antibiotic treatment [,^66^]. Currently, FMT is being explored in numerous clinical trials for treating patients with inflammatory bowel disease (IBD). Results have shown varying degrees of efficacy among patients, which may be attributed to differences in administration methods, such as the route, dosage, and formulation [,[67],^68^]. FMT not only modulates the composition of the gut bacterial microbiota but is also influenced by the gut mycobiota, which can critically affect its therapeutic efficacy. Emerging evidence indicates that the colonization of *Candida albicans* and *Yarrowia* species is significantly associated with diminished FMT effectiveness in the treatment of *Clostridioides difficile* infections [,[69],^70^]. In contrast, FMT responders exhibit enhanced fungal diversity, characterized by a higher relative abundance of beneficial genera such as *Saccharomyces*, *Aspergillus*, and *Penicillium* [,^69^,,^71^]. Collectively, gut fungal communities are closely associated with FMT efficacy, and further mechanistic studies are required to guide the optimization and improvement of FMT treatment outcomes.

### Future perspective

Compared to bacteria, the fungal component of the gut microbiota remains understudied. However, it is worth maintaining optimism about the potential of research on the fungal microbiota to enhance our understanding and treatment of IBD, asthma and other diseases such as carcinogenesis. Emerging evidence also suggests that fungi may also play significant roles in cancer development and progression, particularly through immune modulation and interactions with tumour-associated microbiota [21,,72,,73]. Although beyond the scope of this review, exploring the role of gut and tumour-associated fungi in cancer immunity represents an important future direction. Recent research has identified prevalent colonizers in both humans and mice. In this scenario, strain-specific colonization will provide valuable insights into the immune mechanisms underlying the maintenance of immune homoeostasis and progression of specific inflammatory diseases. Given the diversity of mouse colonies and housing environments, researchers should carefully consider differences in gut microbiota and mycobiota, as these variations may influence immune responses. Large cohort studies could provide valuable insights by incorporating fungal species into broader microbiome research and clarifying the specific influence of fungi. New models such as rewilded mice or humanized gnotobiotic mice may enable studies to be conducted under more realistic conditions close to humans. Future research should focus on elucidating the mechanisms by which gut fungi influence systemic immunity and on developing precision-based approaches to modulate the mycobiome for improved health outcomes.

## Data Availability

No new data generated or analysed
